# Hiding from the Moonlight: Luminosity and Temperature Affect Activity of Asian Nocturnal Primates in a Highly Seasonal Forest

**DOI:** 10.1371/journal.pone.0036396

**Published:** 2012-04-27

**Authors:** Carly Starr, K. A. I. Nekaris, Luke Leung

**Affiliations:** 1 School of Agriculture and Food Science, University of Queensland, Gatton, Queensland, Australia; 2 Oxford Brookes University, Nocturnal Primate Research Group, School of Social Sciences and Law, Oxford, United Kingdom; Texas A&M University, United States of America

## Abstract

The effect of moonlight and temperature on activity of slow lorises was previously little known and this knowledge might be useful for understanding many aspects of their behavioural ecology, and developing strategies to monitor and protect populations. In this study we aimed to determine if the activity of the pygmy loris (*Nycticebus pygmaeus*) is affected by ambient temperature and/or moonlight in a mixed deciduous forest. We radio-collared five females and five males in the Seima Protection Forest, Cambodia, in February to May, 2008 and January to March, 2009 and recorded their behaviour at 5 minutes intervals, totalling 2736 observations. We classified each observation as either inactive (sleeping or alert) or active behaviour (travel, feeding, grooming, or others). Moon luminosity (bright/dark) and ambient temperature were recorded for each observation. The response variable, activity, was binary (active or inactive), and a logit link function was used. Ambient temperature alone did not significantly affect mean activity. Although mean activity was significantly affected by moonlight, the interaction between moonlight and temperature was also significant: on bright nights, studied animals were increasingly more active with higher temperature; and on dark nights they were consistently active regardless of temperature. The most plausible explanation is that on bright cold nights the combined risk of being seen and attacked by predators and heat loss outweigh the benefit of active behaviours.

## Introduction

The sensory world of the forest at night has strongly influenced the behaviour and physiology of nocturnal mammals. In the absence of visual sensory cues, many nocturnal mammals are solitary [1], rely heavily on smell [2], and use crypsis to avoid predators [3]. Consequently, the activity of many nocturnal mammalian prey species is affected by the intensity of nocturnal illumination. Some prey animals may reduce (lunar phobic) or increase (lunar philic) activity with increasing moon luminosity depending on their vulnerability to predators under bright moonlight [4], [5], [6], [7], [8]. Other prey animals may not change their activity in response to moon luminosity (lunar neutrality) [9]. Temperature also affects the activity of nocturnal mammals [10] and also their food supply, with the abundance and activity of invertebrates being lower with decreasing temperature. Periods of torpor and reduced activity are most commonly energy saving adaptations in response to extremely hot or cold climatic fluctuations [11].

Amongst primates, some species are nocturnal specialists, and others are cathemeral, meaning their activities are distributed throughout the 24-hour cycle for at least some parts of the year [12]. Together these comprise some 35% of primate species, found in every part of their range from the Neotropics (night monkeys), Africa (galagos, pottos), Madagascar (lemurs), and Asia (tarsiers, lorises) [13]. All of these taxa are equipped with a suite of morphological traits suited to this mode of life including the presence of a *tapetum lucidum* (a reflecting retinal layer enhancing vision at night) or extra-enlarged eyes to allow in light; a low basal metabolic rate to thermal-regulate on cold nights; and enhanced olfactory and vocal signals to communicate in the dark [14].

Some primate species, through enhanced vision and ability to mob predators, to produce alarm calls and/or move off with speed, are better equipped to detect and avoid predators near and during full moons, and hence, do not need to reduce activity on bright nights [9], [15], [16]. Bright nights may enhance foraging efficiency in some primates, allowing them to see and catch more insect prey and ripe fruits [8]. Recent studies have reported different combinations of species-specific lunar responses in nocturnal primates [9], [17], [18], [19], [20], [21]. The spectral tarsier (*Tarsius spectrum*) and numerous species of galago (*Galago senegalensis, G. moholi, Galagoides demidovii, G. zanzibaricus, Sciurocheirus alleni*, and *Euoticus elegantulus*) increase foraging and/or travelling during bright moon nights [8], [22], [23], [24].

Regulation of body temperature in nocturnal primates has been shown to be of particular importance in one family of Malagasy lemurs (Cherogaleidae) [25], [26], [27], [28], [29], [30]. Torpor and hibernation have been studied extensively in these species, showing some of the most remarkable specialisations of any nocturnal mammal, such as extreme lowering of the body temperature, and heavy storage of fat in the tail [29], [30]. Torpor has also been observed in a captive sub-adult *Galago moholi* [31], although it was not observed in two long-term field studies of this species [15], [21], [32]. Indeed, the opposite was the case, when galagos became active in the daytime to feed on valuable gum resources [15].

The Asian lorises (subfamily Lorisinae) have classically been described to avoid predators by crypsis [33]. Ranging in body size from 120 to 2100 g, loris species are characterized by non-saltatory locomotion [34], moving slowly and deliberately through vegetation [35]. They have reduced second digits, and their limbs have *retia mirabilia*, allowing them to maintain grip for long periods of time [34], [36], so that they can remain utterly still in the presence of a threat. All lorises have monochromatic vision, possessing only a single functional medium/long wavelength-sensitive cone [37], [38], which affects the way they perceive predators and food in open versus dense forests.

This was clearly demonstrated by two species of South Asian slender lorises (*Loris*). *L. lydekkerianus* inhabits open dry acacia scrub forests. On bright nights it increased foraging activity for energy-rich foods, but do not alter their distance travelled [15], [24]. It whistled more frequently during dark nights than bright nights. In contrary, the rain-forest dwelling *L. tardigradus* whistled more frequently on bright nights than dark nights [39]. On dark nights it slept, travelled less and groomed more, reducing active behaviours [39].

The lunar response of slow lorises in South and Southeast Asia has not been previously investigated prior to the present study. Metabolic rates are extremely low in slow lorises [40], [41], [42], [43], with the greater slow loris (*N. coucang*) having a basal metabolic rate 60% lower than predicted [44]. Captive pygmy slow lorises (*N. pygmaeus*) in Northern Vietnam exhibited extensive periods of inactivity and reduced body temperatures during cooler months [45], [46]. This species was also encountered at lower rates during colder months in the dry season in Laos, suggesting its activity was reduced with lower temperatures [47].

We, therefore, chose to study the lunar response of the pygmy slow (hereafter pygmy) loris *Nycticebus pygmaeus*. Endemic to Vietnam, Laos, southern China and eastern Cambodia [48], [49], [50], [51], it is listed as Vulnerable in the IUCN Red List [52], and in Appendix 1 of CITES [53] based on increasing and unsustainable trade [53], habitat loss and degradation [52].

Optimal foraging theory in its most basic form suggests an animal will forage in ways that will maximise its energy intake [54], with a trade off between the risk of being seen by predators on bright nights, and the benefit of increasing food intake. The study site is located in a seasonal deciduous forest rich in species diversity of civets, pythons, monitor lizards and predatory birds, which are known predators of other small nocturnal primates including loris species [55], [56]. The site experiences low temperatures during the dry season from December to February, with a marked reduction of foliage, increasing the visibility for predators. Foraging at low temperatures would increase heat loss, and foraging during bright nights would increase the risk of predation. These conditions allow us to test how moonlight and temperature interact to affect nocturnal animal behavior.

We predict that the pygmy loris will:

1. be more active during dark nights when predation risk decreases;
2. reduce active behaviour such as travel and foraging, and increase inactive behaviours such as resting and grooming during cold periods.

## Results

**Figure 1:**
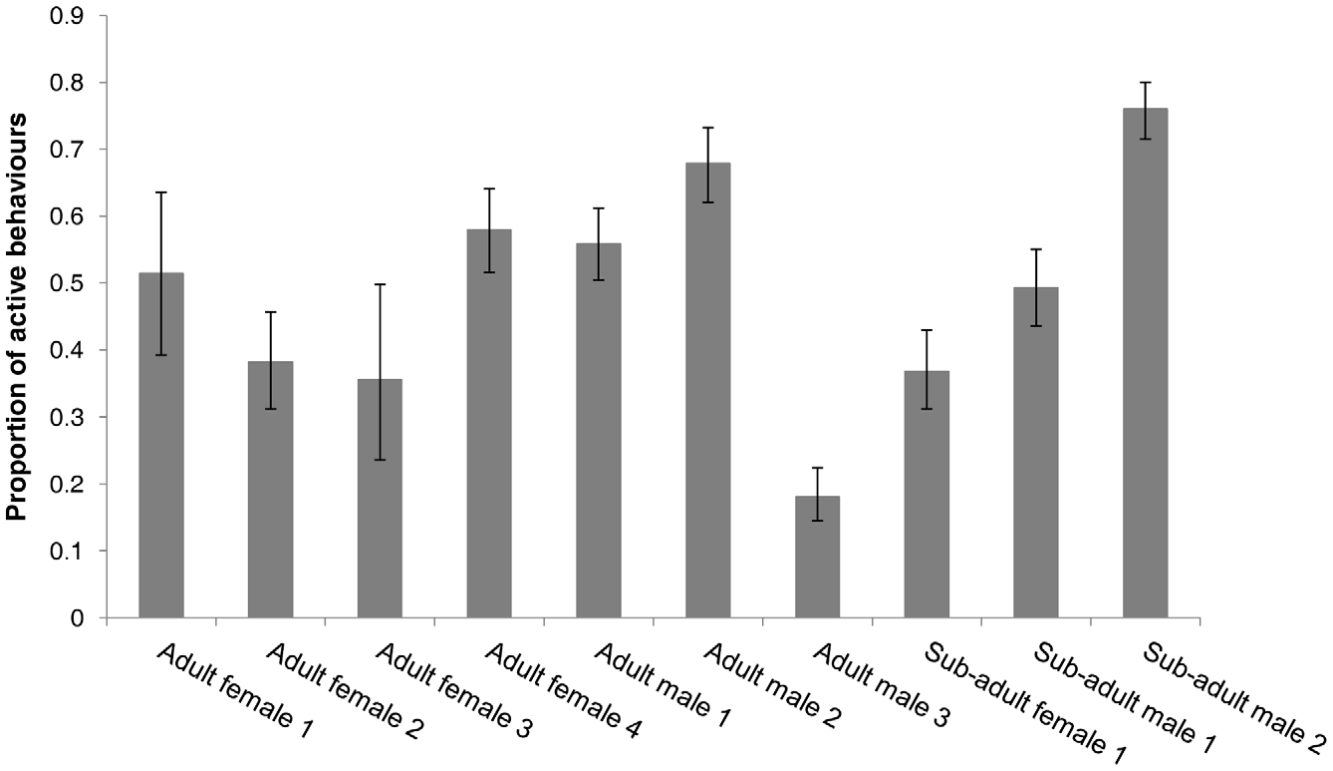
Proportion of active behaviours across individuals in the study. The standard error of the logit values were used to construct 95% confidence intervals indicated by the error bar.

**Figure 2:**
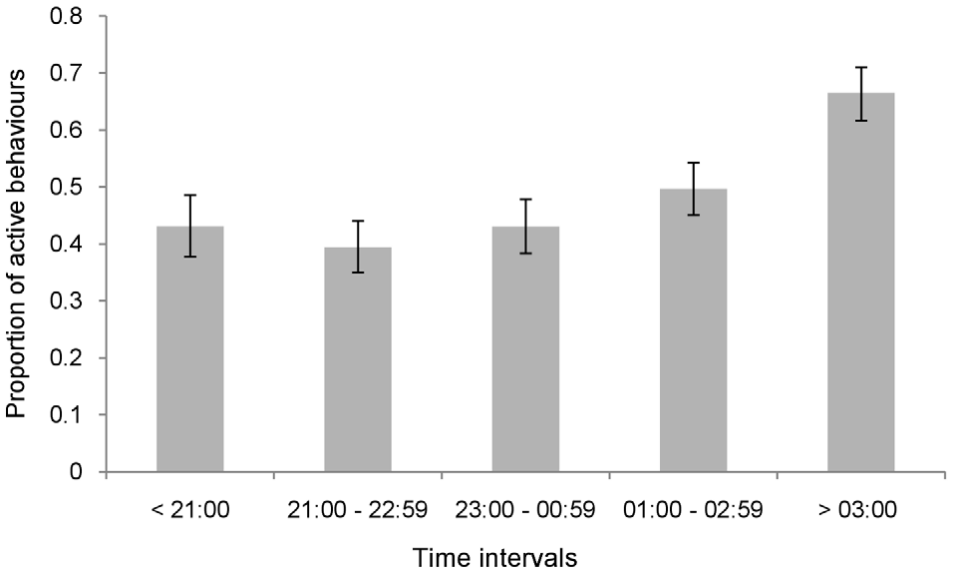
Proportion of active behaviours across the night excluding periods of astronomical twilight. The standard error of the logit values were used to construct 95% confidence intervals indicated by the error bar. Means are adjusted for other fixed effects.

Mean activity differed significantly between the 10 studied individuals (χ^2^ = 234.86, *d.f.* = 9, p = <0.001; Figure 1). Time of the night significantly affected mean activity (χ^2^ = 73.72, *d.f.* = 4, p = <0.001; Figure 2). These individual and temporal differences were accounted for in the following test results.

**Figure 3:**
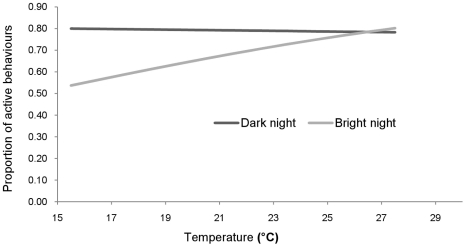
Interaction between moonlight and temperature on activity of the pygmy loris as predicted by the model.

Temperature on its own did not significantly affect mean activity (χ^2^ = 0.06, *d.f.* = 1, p = 0.809). Mean activity was significantly affected by moonlight (χ^2^ = 5.14, *d.f.* = 1, p = 0.023), and the interaction between moonlight and temperature was significant (χ^2^ = 4.05, *d.f.* = 1, p = 0.044), with temperature affecting mean activity during bright nights, but not dark nights (Figure 3).

Mean activity was constant across the temperature range from 16 to 28°C on dark nights, and did not reach the proportion on bright nights until the temperature reached above 26°C.

## Discussion

The observed effect of moonlight on activity indicates that during the dry, cool season the pygmy loris is lunar phobic. A plausible explanation for this is that during this season the deciduous forest is sparse, providing reduced cover for animals from potential predators, resulting in a less effective anti-predator strategy. This was exacerbated by burning of the forest by local people to improve access for collecting non-timber forest products [57]. The pygmy loris uses more crypsis and concealment than faster nocturnal primates such as the slender lorises, galagos, mouse lemurs and tarsiers [9], [17], [19], [20], [21], [24]. The most plausible explanation for the pygmy loris reducing its activity with lower temperature on bright nights is that the combined risk of both predation and heat loss on bright, cold nights outweigh the benefit of active behaviours.

Avoiding predators at the study site requires multiple adaptations, as potential predators occur on the ground (small cats), in the trees (snakes), in the air (hawk-eagles), and those animals that move between these areas (monitor lizards, civets) [58]. Kenyon [pers. comm.] reports that monitor lizards and birds of prey caused high levels of mortality to reintroduced pygmy lorises in Vietnam. Decreasing activity during the light moon would confound at least several classes of these predators.

The lunar phobic behaviour observed is possibly seasonal and the pygmy loris may be more active on bright nights during the wet season when temperatures are higher and the forest provides denser vegetation cover. Other mammals are known to display seasonal variation in their lunar response [5]. Unfavourable conditions due to the lack of rain for tropical plants and invertebrates during the dry season may lower food supply and the pygmy loris may use inactivity to conserve energy in response. The heavy reliance of the species on gum during the study [59] parallels that of *Galago moholi* which relied on this resource during the cold winter months, when it also decreased activity [60].

It has been suggested that highly insectivorous nocturnal primates will be more lunar philic because moonlight improves their hunting success [8], [9], [24]. Although animals in our site were frequently observed to catch and consume arthropods, and a high proportion of invertebrates were found in scats throughout the study period (CS, unpub. data), our data did not indicate that this resource was important enough to select for lunar philia in the local population at the site.

A behaviour exhibited rarely by the study animals was emitting their loud long-distance whistle. Although slender lorises produced this whistle varyingly in dry versus wet environments, they still whistled multiple times per hour regardless of moonlight [24], [39]. Pygmy lorises kept in outdoor enclosures also whistle regularly [46]. Towards the end of our study, the study animals were heard to begin to whistle on occasion, and it might have been that as the forest became denser with foliage they could afford to decrease crypsis.

Ambient temperatures are known to affect the activity in a range of cathemeral [61], [62], [63], [64], [65], [66] and nocturnal [29], [67] primates. Many of these primates undergo periods of torpor or heterothermy to conserve energy to cope with low ambient temperatures [25], [26], [27], [28], [29], [30], [31]. Fernández-Duque et al. [68] reported that the activity of a lunar philic primate was reduced during cooler temperatures, despite luminance. Some species are known to adapt their behaviours as a coping mechanism. For example, at lower temperatures, *G. moholi* displayed diurnal activity such as moving into the sunlight, or feeding during the day [60], [69], [70]. Our study animals became inactive on cool nights only when the moon was up, however it is plausible the species may undergo periods of torpor or heterothermy in cooler parts of their range such as Northern Vietnam, where nightly temperatures are known to reach 5°C [46], [71]. Female pygmy lorises experience late pregnancy, birth and begin lactation during the cool season [72], which would further increase their energy demands, and may result in further inactivity during times of food shortage and low ambient temperatures.

Field surveys have indicated that densities of pygmy lorises in Cambodia are low [73], and an effective monitoring method is required. Our data suggest that monitoring of this species should occur on dark nights, or warm bright nights during the dry season to maximize the chance of encountering animals. Enforcement initiatives may also focus on patrolling the forest during these nights when pygmy lorises are more active, and, therefore, more likely to be encountered by poachers.

## Methods

### Ethics statement

This research involved work with wild non-human primates. Animal ethics approval for this project (Approval number: SAS/696/07/PhD) was approved by the University of Queensland Animal Ethics Committee. All experiments comply with the current laws of the country where they were performed.

Animals were hand captured, fitted with a radio-collar and immediately released at the point of capture in the field to minimise stress in accordance with the recommendations of the Weatherall report, “The use of non-human primates in research”. Direct observations of study animals were used to collect behavioural data, and we attempted to retrieve all radio-collars on completion of the project to minimise discomfort of the study animals. We were unable to recapture 2 individuals despite numerous attempts; however the collars were made of thin leather bands which would eventually break free from the animals.

### Study site

**Figure 4:**
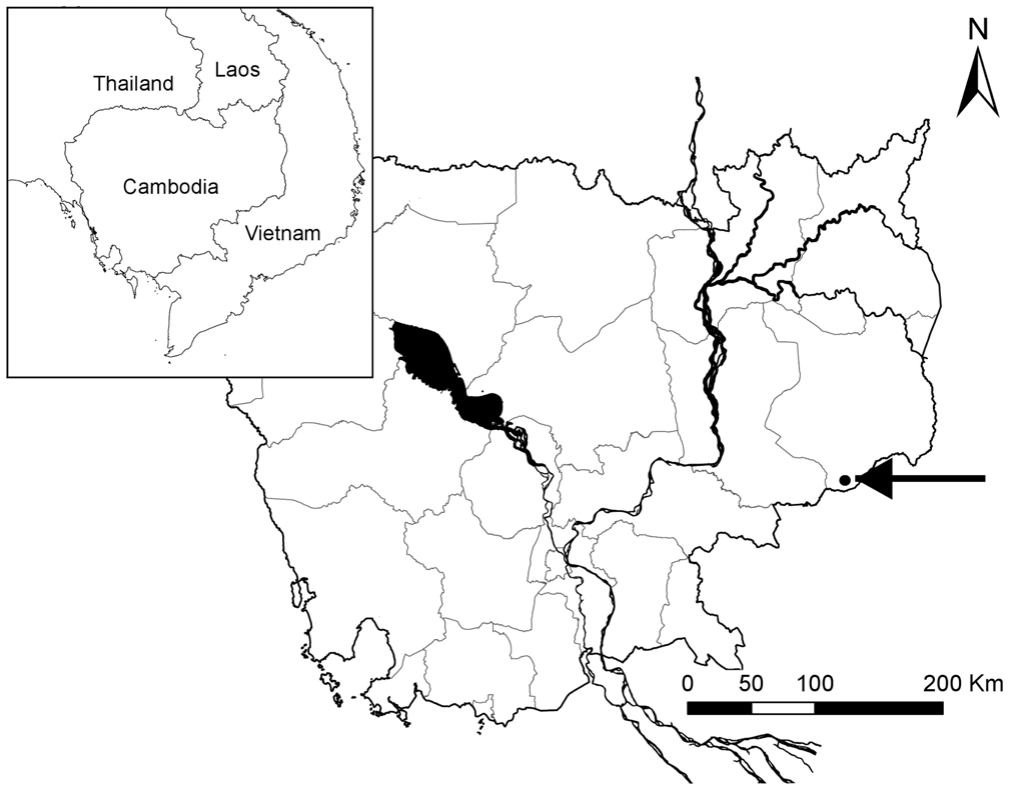
Location of Seima Protection Forest, Cambodia. The study site is indicated on the map.

The study was conducted in the Seima Protection Forest (PF), in southern Mondulkiri province, Cambodia (Figure 4). This conservation area encompasses 292,690 hectares. The study was conducted in two periods: from 12 February to 31 May 2008; and from 9 January to 24 March 2009.

In Mondulkiri dry season extends from November to April and the rainy season from May to October and the mean annual rainfall is approximately 2000–2500 mm [74]. Rainfall levels in the southern, more mountainous part of the province are considerably higher, with an annual mean of over 3,200 mm. The conservation area lies between 100–700 m asl on the western slopes of the Sen Monorom plateau, and in the south is part of the Annamite Range [57].

The Seima PF consists of a mosaic of forest types, including semi-evergreen, mixed deciduous, deciduous dipterocarp, and evergreen forests [75]. The mixed deciduous forest is dominated by *Lagerstroemia sp.*, a deciduous tree species. This study occurred in mixed deciduous forest, which had patches of bamboo throughout. We selected this forest type for our study for two reasons. First, spotlight surveys identified higher encounter rates in this habitat type [73], and second, it was easier to catch, collar and observe animals in mixed deciduous forests as they were less dense, making it easier to pursue animals quietly. We were unable to collect data during the wet season due to heavy rains and inaccessibility to the site during these months.

### Study animals

Animals were located using Petzl Zoom 4.5v headlamps (Petzl, Crolles, France) with a red filter and hand captured. Each captured animal was fitted with a two-stage transmitter (Sirtrack®, Havelock North, New Zealand) and released at the point of capture. Ten individuals were tracked on foot, using a 6-element Yagi antenna (Sirtrack®, Havelock North, New Zealand), and a portable radio receiver (ICOM IC-R10 receiver; Icom Inc. ®, Osaka, Japan). Data were collected on nightly follows of collared animals between dusk and dawn, over two shifts: 18:00–00:00 and 00:00–06:00. Each individual had at least one all-night follow from 18:00–06:00. We captured and collared four adult females, three adult males, one sub-adult female, and two sub-adult males. Sub-adults were generally smaller, and were recognizable as observed by Wiens and Zitzmann [76] as having teeth that were white and unworn, little or no wear on the inner surfaces of nails, longer, and paler fur when compared to adult animals, and females were nulliparous.

### Behavioural observation

Focal animal sampling and instantaneous sampling methods were used to collect behavioural data [77], [78], and scan samples were taken at 5-min intervals, recording data at the end of each observation [78], [79]. We adapted behavioural categories from Gursky [8] and Nekaris [80]. Our categories were alert (eyes were open, the animal was not moving), sleeping (eyes were closed), travel, feed (we pooled data on hunting and foraging in to this category), activity unknown, auto-grooming, and others (less common behaviours such as scent marking and allo-grooming were pooled in to this category). Very few observations were made in the category ‘others’. A total of 2736 (1233 active, 1503 inactive) observation points were collected from 10 individuals. The number of observations made on bright and dark nights are listed for individual study animals in Table 1.

**Table 1:** Number of observations collected on bright and dark nights for individuals.

Individual ID	Bright nights	Dark nights
Adult female 1	11	54
Adult female 2	110	76
Adult female 3	174	78
Adult female 4	164	104
Adult male 1	105	245
Adult male 2	166	153
Adult male 3	411	113
Sub-adult female 1	152	123
Sub-adult male 1	50	60
Sub-adult male 2	165	222

### Analysis

**Figure 5:**
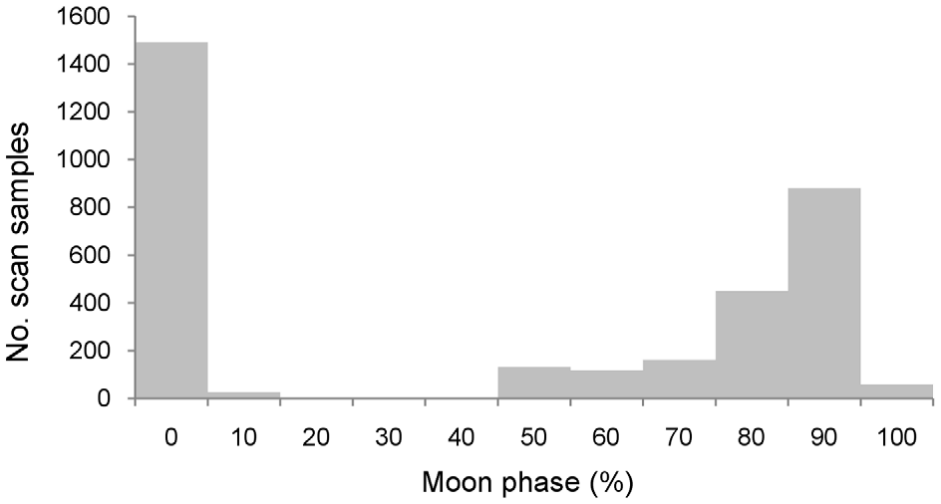
Frequency distribution of observations across moon phases. Moon phase is expressed as the illuminated percentage of the portion of moon. When the moon was not above the horizon the moon phase was given a value of 0.

The response variable, activity, was binary, and a logit link function was used. The model allowed baseline activity to vary between individuals. Inactive behaviour was either sleeping or being alert. All other categories of behaviours were defined as active behaviour. The model consisted of individual ID, time of night, temperature, moonlight and the interaction between temperature and moonlight. Time of the night was classified (taking into account astronomical twilight) by five time bands: <21:00; 21:00–22:59; 23:00–00:59; 01:00–02:59; and >03:00. Observations collected during the twilight periods were excluded from the analysis to avoid the possible effect of sunlight. Nearly all observations were collected when the moon was either below the horizon and had a phase value of 0% (dark nights), or a phase value from 50% to 100% (bright nights). Preliminary analyses of the data indicated that classifying moonlight by dark/bright nights was more parsimonious than using an illumination index given nearly all observations were collected on either dark or bright nights (Figure 5). Illumination indices were used in some previous studies of primate activity [81], [82]. This index was used in earlier models during preliminary analysis but was not used in the final model to improve the parsimony of the model. Given the small number of individuals observed, we did not investigate the effect of sex or age class on activity. The analyses were performed using the GENMOD Procedure in SAS® version 8.2.

### Temperature

**Figure 6:**
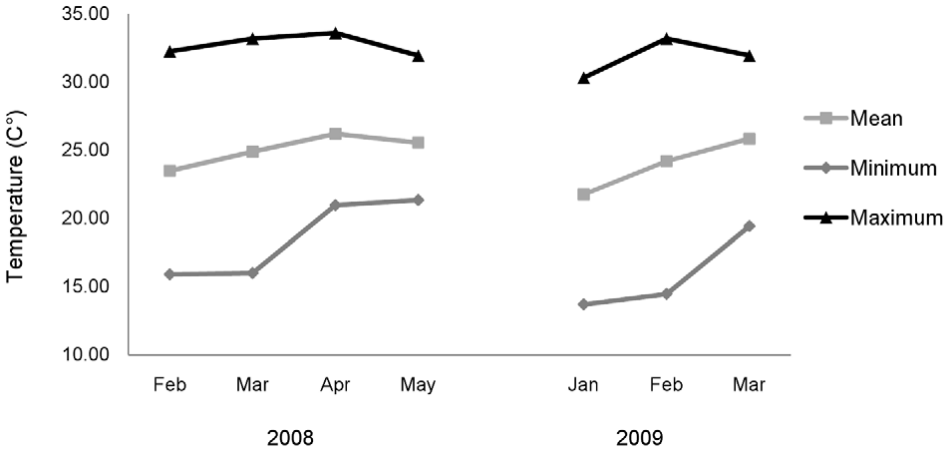
Mean, minimum and maximum nightly temperatures in the Seima Protection Forest during the study period.

Ambient temperature was collected by a HOBO Pro series data logger (Onset®, Massachusetts, US) at the Seima PF base camp. The logger was set to record at 15 minutes intervals. Linear interpolation was used to calculate temperature for 5 minute intervals. Temperature fluctuated between dusk and dawn across the study period (Figure 6).

### Moon

Sunrise, sunset, moonrise, moonset, and moon phase were sourced from Geoscience Australia (http://www.ga.gov.au/geodesy/astro/) for the time at which each observation was collected.
